# Effect of traditional Chinese medicine-based rehabilitation nursing combined with scalp acupuncture on negative emotions and quality of life of patients with stroke: A randomized controlled trial

**DOI:** 10.1097/MD.0000000000031330

**Published:** 2022-10-28

**Authors:** Jingjun Xie, Jinxia Li, Qi Sun, Jianli Cai

**Affiliations:** a Department of Acupuncture, The First People’s Hospital of Huzhou, Huzhou, Zhejiang Province, China; b Department of Acupuncture, Huzhou Hospital of Traditional Chinese Medicine Affiliated to Zhejiang Chinese Medical University, Huzhou, Zhejiang Province, China; c Nursing Management Department, The First People’s Hospital of Huzhou, Huzhou, Zhejiang Province, China.

**Keywords:** negative emotion, quality of life, scalp acupuncture, stroke, traditional Chinese medicine-based nursing

## Abstract

This study aimed to analyze the effect of traditional Chinese medicine-based rehabilitation nursing combined with scalp acupuncture (TCMRN + SA) on negative emotions and the quality of life of patients with stroke.

**Methods:**

102 patients with stroke admitted to The First People’s Hospital of Huzhou from September 2019 to December 2020 were included in this study using the convenience sampling method and split into an observation group and a control group at random (n = 51 in each group). Individuals in the control group received TCMRN, whereas patients in the observation group received TCMRN + SA. Furthermore, the negative emotions and quality of life of the individuals in both groups were evaluated before and after the intervention using the Pittsburgh sleep quality index scale, Self-Rating Depression Scale (SDS), Self-Rating Anxiety Scale (SAS), as well as Activity of Daily Living Scale. Furthermore, the efficiency of the sleep-quality intervention between the 2 groups was compared.

**Results:**

After the intervention, the Pittsburgh sleep quality index scale, SDS, as well as self-rating anxiety scale scores of individuals in the observation group were considerably lower in comparison to the individuals in the control group (*P* < .01). Activity of daily living scale scores in the observation group also differed considerably from those in the control group (*P* < .01). Moreover, the sleep quality efficiency rate in the observation group (90.19% [46/51]) was substantially higher than that in the control group (70.59% [36/51]) (*P* < .05).

**Conclusion:**

TCMRN + SA can effectively improve patients’ negative emotions and quality of life and is worthy of clinical promotion and application.

## 1. Introduction

Stroke is a chronic condition with high clinical morbidity and disability. Most individuals with stroke suffer from physical dysfunction, along with physical and psychological disorders such as sleep disturbance, anxiety, and depression.^[1]^ Delayed stroke treatment can further affect patients’ neural functional recovery and quality of daily life, and can even increase the risk of stroke recurrence. Traditional Chinese medicine-based rehabilitation nursing (TCMRN), guided by the basic theories of traditional Chinese medicine (TCM), promote the recovery of patients’ bodily functions by regulating qi and blood and dredging main and collateral channels.^[2]^ Scalp acupuncture (SA), one of the treatment methods of traditional acupuncture, is an effective therapy to treat diseases by stimulating specific areas in the hairline region of the head and has now become a prevalent method for treating the sequelae of stroke, with the advantages of easy operation and easy-to-find acupuncture points.^[3,4]^ In our investigation, we studied the effect of TCMRN + SA on negative emotions and the quality of life of patients with stroke.

## 2. Material and Methods

### 2.1. Patient selection and general information

In this single-blind, exploratory, randomized, controlled study 102 patients with stroke treated in our hospital from September 2019 to December 2020 were assessed for eligibility and recruited. All eligible patients were randomized at a ratio of 1:1 via the random envelope method to either an observation group or a control group. The patients and their families were informed about the purpose and significance of the study before the study and signed the informed consent form. This study was reviewed and approved by the medical ethics committee of our hospital.

Following were the inclusion criteria for this study: stroke determined by the diagnostic criteria of the Academic Conference on Stroke,^[5]^ supported by cranial CT (or MRI) imaging evidence; age 40 to 70 years; disease course within 6 months; and informed consent form signed by patients or their families. Following were the exclusion criteria for this study: severe heart, liver, kidney, and other organ diseases; long-term use of psychotropic drugs or sedatives; and noncompliance of patients or their families. The First People’s Hospital of Huzhou City’s medical ethics committee granted its approval for this study (approval No. 2021KYLL019).

### 2.2. Methods

The intervention team was established before this study, consisting of 1 doctor each from the neurology and acupuncture departments, one rehabilitation technician, and 4 specialist nurses.

#### 2.2.1. Control group.

Individuals in the control group received TCMRN, including TCM-based emotional nursing, TCM-based diet nursing, and TCM-based rehabilitation: TCM-based emotional nursing: TCM-based emotional nursing originated from the *HuangdiNeijing*.^[6]^ Patients were provided targeted psychological counseling based on TCM theories. In addition, we explained the relationship between the 7 human emotions and internal injuries in TCM to further guide patients on self-control of their emotions, or suggested patients listen to soft music before bed to nourish their minds and improve low mood; TCM-based diet nursing: Patients receive dialectical diet training under the guidance of TCM theory. In addition, they were ensured 3 regular meals, appropriate food pairing with the “5 tastes” of “cold” and “warmth”, and encouraged to eat foods with medicinal properties; TCM-based rehabilitation: We instructed the good limb position and the motor training of the affected limb function and administered patients with back-patting or massage along channels (once/d). In addition, local pressing with beans on auricular points Shen Men, Heart, Sympathetic, and Subcortical acupoints were performed (3–5 times/d, approximately 3 min each time), followed by acupoint patching (lasting 2–4 d). Redness, heat, and swelling of the ears suggested the appropriate level of pressure. Finally, we performed 4-week nursing education.

#### 2.2.2. Observation group.

Patients in the observation group received TCMRN + SA. Specifically, disposable filiform needles (0.25 mm × 40 mm) procured from Suzhou Wuzhong District Dongfang Acupuncture Instrument Factory were used for SA according to the international standardized protocol for head acupuncture and brain functional region localization.^[7]^ Specifically, the frontal midline, the healthy anterior parieto-temporal oblique line, and the posterior parieto-temporal oblique line were acupunctured. In addition, the point-through-point method was used and the needle tip was directed downward at 15° to 20° to the scalp. We performed twirling of the needle first, followed by lifting ‐ inserting. In addition, the needle was retained for 1 hour after needling response, during which twirling of the needle was performed every 30 minutes (once/d for 4 weeks).

### 2.3. Outcome measures

(1) Pittsburgh Sleep Quality Index scale (PSQI)^[8]^ covers 7 items (sleep latency, hypnotic drug use, sleep duration, daytime dysfunction, sleep efficiency, sleep quality, and sleep disturbance). The scale has a score range of 0 to 21, with higher scores suggesting poorer sleep quality.
(2) Emotional status was evaluated by the Self-Rating Anxiety Scale (SAS) and Self-Rating Depression Scale (SDS) developed by Zung.^[9]^ Each scale has 20 items, and scores below 50 are considered normal, whereas scores ≥ 50 indicate the presence of significant anxiety or depression (higher scores indicate more severe anxiety and depression).
(3) Activity Of Daily Living Scale (ADL)^[10]^ involves multiple motions such as going up and down stairs, eating, excreting stool and urine, and dressing. The total score of this scale is 100, with higher scores indicating patients’ stronger ability to live.
(4) Sleep efficiency assessment criteria in this study were based on the *Guidelines for the Clinical Research of Chinese Medicine New Drugs* and “Nimodipine Method”^[11]^ as follows: clinical cure: normal sleep latency or sleep duration ≥ 6h at night, deep sleep, energetic status after waking up, and score reduction ≥ 95%; markedly effective: significantly improved sleep quality, sleep duration increased ≥ 3h, increased sleep depth, and score reduction ≥ 70%; effective: increased sleep duration but < 3h and score reduction ≥ 30%. Ineffective: no significant improvement in sleep duration, or even worsening, and score reduction < 30%. Total effective rate = (clinically cure + markedly effective + effective)/ total cases × 100 %.

### 2.4. Statistical analysis

SPSS22.0 was used for data analysis. Measurement data were expressed as mean ± standard deviation $\left(\bar{x}\pm \;s\right)$, and a t-test was used for comparison between groups. Enumeration data were expressed by frequency, and the *χ*^2^ test was used for comparison between groups. In addition, *P* < .05 indicated a significant difference.

## 3. Results

### 3.1. Comparison of general information between the 2 groups

Finally, 51patients in the control group and 51 patients in the observation group completed the study. There was no significant difference in terms of clinical data, including age, sex, hemiparesis side, type of stroke and course of disease between the 2 groups (*P* > .05), as shown in (Table 1)

**Table1:** Comparison of general information between the 2 groups.

Group	Case	Sex (n)		Type of stroke (n)		Hemiparesis side (n)		Age (years, $\bar{x}\pm s$ )	Disease course (d, $\bar{x}\pm s$
		Men	Women	Intracerebral hemorrhage	Cerebral infarction	Left	Right		
Observation group	51	26	25	23	28	13	38	53.20 ± 5.63	42.54 ± 7.34
Control group	51	24	27	21	30	15	36	52.42 ± 4.55	41.25 ± 6.53
*t*/*χ*^2^		0.282		0.276		0.358		0.263	0.168
*P*-value		.602		.587		.648		.769	.872

### 3.2. Comparison of PSQI scores for each item and total scores before and after intervention between the 2 groups

After the intervention, the PSQI scores of individuals in the observation group were lower in comparison to the individuals in the control group (*P* < .01). (Table 2)

**Table 2:** Comparison of PSQI scores for each item and total scores between the 2 groups ($\bar{x}\pm s$).

Group	Case	Sleep quality	Sleep latency	Daytime dysfunction	Sleep duration	Sleep efficiency	Sleep disturbance	Hypnotic drug use	Total PSQI score
Before intervention									
Observation group	51	1.62 ± 0.92	1.34 ± 1.01	1.68 ± 0.88	1.41 ± 1.01	1.24 ± 0.98	1.54 ± 0.86	1.58 ± 0.78	9.99 ± 0.85
Control group	51	1.61 ± 0.94	1.31 ± 0.98	1.55 ± 0.99	1.39 ± 0.97	1.22 ± 0.89	1.53 ± 1.01	1.61 ± 0.92	10.02 ± 0.67
*T*-value		0.368	0.453	0.379	0.447	0.496	0.561	–0.527	–0.207
*P*-value		>.05	>.05	>.05	>.05	>.05	>.05	>.05	>.05
After intervention									
Observation group	51	0.75 ± 0.85	0.68 ± 0.59	0.81 ± 0.64	0.68 ± 0.60	0.73 ± 0.57	0.78 ± 0.65	0.72 ± 0.68	4.36 ± 1.16
Control group	51	1.29 ± 0.93	1.12 ± 0.83	1.26 ± 0.79	0.98 ± 0.90	1.15 ± 0.63	1.19 ± 0.75	1.18 ± 0.67	6.42 ± 1.90
*T*-value		–2.214	–2.082	–2.136	–2.096	–2.153	–2.172	–2.161	–6.605
*P*-value		<.05	<.05	<.05	<.05	<.05	<.05	<.05	<.01

PSQI = Pittsburgh Sleep Quality Index Scale.

### 3.3. Comparison of SAS, SDS, and ADL scores before and after intervention between the two groups

After the intervention, the SDS, as well as SAS scores of individuals in the observation group were lower in comparison to the individuals in the control group (*P* < .01).ADL scores in the observation group also differed considerably from those in the control group (*P* < .01). (Table 3)

**Table 3:** Comparison of SAS, SDS, and ADL scores between the 2 groups.

Group	Case	SAS	SDS	ADL
Before intervention				
Observation group	51	59.78 ± 4.44	59.64 ± 4.15	46.57 ± 5.98
Control group	51	58.98 ± 4.22	59.88 ± 0.91	47.10 ± 6.47
*T*-value		0.937	-0.295	-0.429
*P*-value		>0.05	>0.05	>0.05
After intervention				
Observation group	51	40.92 ± 3.40	40.55 ± 3.41	70.65 ± 7.56
Control group	51	50.69 ± 2.64	51.00 ± 3.67	61.37 ± 9.43
*T*-value		–16.198	–14.888	5.482
*P*-value		<.01	<.01	<.01

ADL = Activity of Daily Living Scale, SAS = Self-rating Anxiety Scale, SDS = Self-rating Depression Scale.

### 3.4. Comparison of sleep efficiency after intervention between the two groups

**Figure 1.**
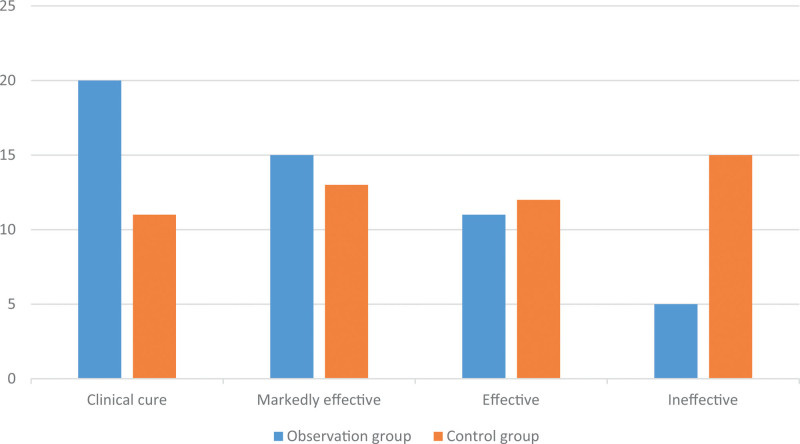
Comparison of sleep efficiency between the 2 groups. The observation group had 20 cases of clinical cure, 15 cases of markedly effective, 11 cases of effective, and 5 cases of ineffective. The control group had 11 cases of clinical cure, 13 cases of markedly effective, 12 cases of effective, and 15 cases of ineffective.

After treatment, the observation group had 20 cases of clinical cure, 15 cases of markedly effective, 11 cases of effective, and 5 cases of ineffective, with a total efficacy of 90.19%. The control group had 11 cases of clinical cure,13 cases of markedly effective, 12 cases of effective, and 15 cases of ineffective, with a total efficacy of 70.59%. The sleep efficiency rate in the observation group (90.19%) was significantly substantially higher than that in the control group (70.59%) (*P* < .05). It shows that TCMRN + SA can effectively improve the sleep quality of patients. (Table 4, Fig. 1)

**Table 4:** Comparison of sleep efficiency between the 2 groups.

Group	Case	Clinical cure (n)	Markedly effective (n)	Effective (n)	Ineffective (n)	Total effective rate (%)
Observation group	51	20	15	11	5	90.19
Control group	51	11	13	12	15	70.59
*χ* ^2^						4.568
*P*-value						<.05

## 4. Discussion

Stroke, one of the highly disabling and fatal diseases in China in recent years, causes neurological and physical dysfunction, which poses a great disturbance to patients’ life and work.^[12]^ At present, in addition to conventional rehabilitation therapies, drugs, especially psychotropic drugs, are primarily used to treat stroke-related complications such as sleep disturbance, mood disorders, and physical dysfunction; however, the long-term use of these drugs is prone to drug resistance.^[13]^ According to TCM theory, stroke is primarily caused by emotional depression and anger, improper diet, and excessive exertion. In this study, we developed the TCMRN + SA scheme based on TCM theory to accelerate the recovery of patients, which is of great significance in clinical settings.

Based on the holistic concept of TCM and the dialectical nursing theory, TCMRN utilizes traditional nursing methods to care for patients with the aid of rehabilitation medical tools, traditional rehabilitation training, and health-preserving methods. SA, a treatment method developed based on traditional acupuncture, is widely used in specific functional areas of the head to prevent and treat disease and has become an effective therapy for treating stroke-related dysfunction.^[14]^ The present study observed significant differences in sleep-quality-related item scores and total scores, sleep-quality intervention effects, and ADL scores between the observation group and the control group (*P* < .05), indicating that TCMRN + SA can improve negative emotions and quality of life in patients with stroke. According to TCM theory, the pathogenesis of stroke is the interaction of wind, fire, phlegm, stasis, and deficiency. The upwelling of blood to the brain and the lack of spirit preservation leads to emotional disorders, stagnation of the channels, and hemiplegia. Overall, these established findings provide the TCM theoretical basis for the use of TCMRN + SA.

In this study, TCM-based emotional nursing improved the effect of emotional counseling by identifying the type of emotional abnormalities in patients and taking targeted counseling measures. By ensuring the patient’s proper diet and the consumption of foods with medicinal properties, the patient’s physique was significantly improved. The back-patting and massage along channels promoted the smooth flow of stagnant meridians, improved blood circulation in the limbs, and enhanced life activities, thereby improving the patients’ quality of life. In addition, the stimulation of the Shen Men, Heart, Sympathetic, and Subcortical acupoints in the ear effectively regulated the function of the internal organs of the patients, reduced the foci of excitation in the cerebral cortex, achieved a balance of yin and yang, alleviated negative emotions in the patients, thereby improving sleep efficiency and quality of life.^[15]^ SA targeting the corresponding functional regions^[16]^ increased the excitability and sensitivity of neuronal cells in the cerebral cortex, promoted the recovery of brain cells in the reversible damage zone and damaged nerve cells, and accelerated the recovery of limb function in patients, thereby improving the quality of life of patients with stroke.

In summary, the optimized treatment scheme–TCMRN + SA can effectively improve the negative emotion and quality of life of patients with stroke. In addition, it has the advantages of high safety, good patient compliance, and few side effects, and is worthy of clinical promotion. However, this study has some limitations due to the lack of long-term follow-up data. Therefore, more in-depth studies are needed to provide a further theoretical basis for clinical research.

## Author contributions

**Conceptualization:** Jing-Jun Xie, Jin-Xia Li.

**Data curation:** Jing-Jun Xie, Qi Sun.

**Formal analysis:** Jing-Jun Xie, Jin-Xia Li.

**Investigation:** Jing-Jun Xie,Jian-Li Cai

**Project administration:** Jin-Xia Li.

**Resources:** Jing-Jun Xie, Qi Sun, Jian-Li Cai.

**Supervision:** Jin-Xia Li.

**Validation:** Jin-Xia Li.

**Writing ‐ original draft:** Jing-Jun Xie.

**Writing ‐ review & editing:** Jing-Jun Xie, Jin-Xia Li.

## References

[1] Jiang H Chen C Hao X. Intervention effect of motor guided imagery training on anxiety, depression and quality of life in stroke patients. Chin J Rehabilit Med. 2020;35:738–40.

[2] Zhu Y. Research on the effect of traditional Chinese medicine nursing technology on reducing postoperative complications in breast cancer patients. Chin J Nurs. 2017;52:289–92.

[3] Lee SH Lim SM. Acupuncture for insomnia after stroke: a systematic review and meta-analysis. BMC Complement Altern Med. 2016;16:228. 27430619 10.1186/s12906-016-1220-z PMC4950252

[4] Zhou JW Li J Zhao JJ. Meta analysis on ischemic stroke treated with scalp acupuncture. World J Acupunct Moxibustion. 2013;23:41–7.

[5] Li Y Wang Z. Minutes of the 12th academic meeting of the neurology professional committee of the Chinese association of integrative medicine. Chin J Integr Med. 2017;3:376.

[6] Xue D Kang D Liu Q. Analysis on the application of TCM emotional nursing in geriatric nursing from 2005 to 2015. Nurs Res. 2016;30:2656–8.

[7] Kim E Kim K. Effects of purposeful action observation on kinematic patterns of upper extremity in individuals with hemiplegia. J Phys Ther Sci. 2015;27:1809–11. 26180326 10.1589/jpts.27.1809 PMC4499989

[8] Fontes F Goncalves M Maia S. Reliability and validity of the Pittsburgh sleep quality index in breast cancer patients. Support Care Cancer. 2017;25:3059–66. 28455545 10.1007/s00520-017-3713-9

[9] Yue T Li Q Wang R. Comparison of Hospital Anxiety and Depression Scale (HADS) and Zung Self-Rating Anxiety/Depression Scale (SAS/SDS) in Evaluating Anxiety and Depression in Patients with Psoriatic Arthritis. Dermatology. 2020;236:170–8. 31434087 10.1159/000498848

[10] Lin L Xu L Su L. Influence of daily life training in the ward on caregivers of stroke patients. Chin J Rehabilit Med. 2020;10:1240–3.

[11] Zheng X. Guidelines for Clinical Research of New Chinese Medicines. Beijing, China: China Pharmaceutical Science and Technology Press, 2002:381–3.

[12] Han Z Chen W Zhou X. Effects of interactive head acupuncture on the balance function of spastic hemiplegia after stroke. Chin J Rehabilit Med. 2018;33:948–52.

[13] Tang L Ma C You F. Effects of low-frequency electrical acupoint stimulation on plasma 5-HT and NE in patients with post-stroke insomnia. China Acupunct. 2015;35:763–7. 26571887

[14] Wang J Pei J Khiati D. Acupuncture treatment on the motor area of the scalp for motor dysfunction in patients with ischemic stroke: study protocol for a randomized controlled trial. Trials. 2017;18:287. 28633675 10.1186/s13063-017-2000-x PMC5479040

[15] Yang P Gu J Chang J. Study on the improvement of the health of the elderly by ear acupoint pressing and moxibustion. Jiangxi Trad Chin Med. 2018;426:59–62.

[16] Chen L Fang J Chen L. The achievements and enlightenment of modern acupuncture therapy based on neuroanatomy in the treatment of stroke. Acup Res. 2014;39:164–8. 24818503

